# Acidification does not alter the stable isotope composition of bone collagen

**DOI:** 10.7717/peerj.13593

**Published:** 2022-06-14

**Authors:** Tess Wilson, Paul Szpak

**Affiliations:** 1 Department of Anthropology, Trent University, Peterborough, Ontario, Canada; 2 Department of Biochemistry and Biomedical Sciences, McMaster University, Hamilton, Ontario, Canada

**Keywords:** Collagen, Deamidation, Demineralization, Hydrochloric acid, Stable isotope analysis

## Abstract

In this study, we compared the elemental and isotopic composition of modern and ancient bone samples pre-treated using different demineralization agents with acidic and neutral pH. The purpose of our research was to examine if demineralization using a mineral acid such as hydrochloric acid (HCl) significantly alters the *δ*^15^N and *δ*^13^C values of bone collagen. Evidence from the elemental and amino acid composition of the samples were incorporated alongside isotopic compositions to provide a holistic view of the effect of demineralization agents on the composition of bone collagen. The stable isotope compositions of collagen extracts were also compared against equivalent whole bone samples to assess whether whole bone has a stable isotope composition that is comparable to collagen demineralized with a neutral agent. Our results demonstrate that bone demineralization using either ethylenediaminetetraacetic acid (EDTA) or HCl yields collagen extracts with stable isotope compositions that are not significantly different, indicating that mineral acid does not alter *δ*^15^N and *δ*^13^C values of bone collagen. The results comparing whole bone and extracted collagen stable isotope compositions indicate that whole bone cannot be used as an effective replacement for bone collagen due to the significantly different stable isotope compositions between these sample materials. In ecological and archaeological studies performing stable isotope analysis on bone, sample pre-treatment to isolate collagen is a necessity to obtain the most reliable and reproducible isotopic measurements.

## Introduction

Stable isotope analysis of animal and human remains from both ancient and modern contexts often use bone collagen as the primary substrate for analysis. To isolate the collagenous fraction of the bone, other components such as lipids, non-collagenous proteins, and bioapatite must first be removed (Longin, 1971). These constituents differ in their elemental and stable isotope composition relative to pure collagen, therefore they must be removed *via* chemical pre-treatment in order to produce accurate results that reflect the true composition of bone collagen (Table 1). The pattern of dietary routing also differs in the various bone components. For example, the carbon portion of lipids and bioapatite are derived from the consumer’s whole diet, while the carbon portion of collagen is derived primarily from the proteinaceous component of the diet (Ambrose & Norr, 1993; Howland et al., 2003). As a result of these different dietary sources of carbon and nitrogen, the stable isotope values are systematically offset between the different bone components (Table 1).

**Table 1:** Relative isotopic and elemental composition of the major bone components.

	*δ* ^**13**^ **C**	*δ* ^**15**^ **N**	**wt% C**	**wt% N**	**Reference**
**Protein**	Intermediate	High	Intermediate	High	Sullivan & Krueger (1981) Herring (2012)
**Bioapatite**	High	n/a	Low	Negligible	Sullivan & Krueger (1981)
**Lipids**	Low	n/a	High	Negligible	DeNiro & Epstein (1977) Herring (2012)

The demineralization process, often performed with a mineral acid such as HCl, separates the inorganic component of the bone into solution thereby isolating the organic fraction (Longin, 1971). Although the demineralization treatment is commonly used for stable isotope research, some have suggested that strong acid demineralization may skew the stable isotope composition of bone protein (Bas & Cardona, 2018; Bas et al., 2019b; Carrasco et al., 2018). Given the potential for uneven loss of organic material during the acidification process, *via* acid hydrolysis of peptide bonds or other mechanisms, samples demineralized in strong acid may have isotopic compositions that do not reflect the true composition of bone collagen (Collins et al., 2002; Schlacher & Connolly, 2014).

The optimization of demineralization protocols and the comparison of different pre-treatment methods has been previously explored (*e.g.*, Carrasco et al., 2018; Collins & Galley, 1998; Jørkov, Heinemeier & Lynnerup, 2007; Sealy et al., 2014; Tatsch, Secchi & Botta, 2016; Tuross, 2012). These studies, however, often compare methodologies that vary in multiple aspects of their pre-treatments, making it impossible to unambiguously identify causation (*e.g.*, Jørkov, Heinemeier & Lynnerup, 2007; Sealy et al., 2014), or lack robust sample sizes (*e.g.*, Carrasco et al., 2018; Tatsch, Secchi & Botta, 2016; Tuross, 2012). Further data are, therefore, required to elucidate the effects of specific aspects of pre-treatment on the stable isotope composition of bone collagen. Several of these studies have purported that demineralization using HCl may alter the *δ*^15^N and/or *δ*^13^C values of bone collagen (Bas & Cardona, 2018; Bas et al., 2019b; Carrasco et al., 2018; Turner Tomaszewicz et al., 2015), suggesting that the alteration of collagen stable isotope compositions is the result of peptide bond cleavage causing the loss of certain amino acids (Bas et al., 2019b). Alternatively, it has been suggested that the leaching of less stable non-collagenous proteins may be uneven in different samples depending on the treatment conditions and preservation of the sample (Bas & Cardona, 2018). Variability in the amount of non-collagenous proteins remaining in the sample (Wadsworth & Buckley, 2018) could result in different stable isotope and elemental compositions for the presumed “pure” collagen sample given that collagenous and non-collagenous proteins differ in their amino acid compositions (Guiry & Szpak, 2020).

In order to test the hypothesis that HCl demineralization alters the stable isotope composition of bone collagen, the stable carbon and nitrogen isotope compositions of whole bone and collagen samples have been compared (Bas & Cardona, 2018; Bas et al., 2019b; Carrasco et al., 2018; Tatsch, Secchi & Botta, 2016; Turner Tomaszewicz et al., 2015). This has led to the suggestion that rather than pre-treating samples *via* demineralization, whole bone samples should be used in place of collagen to prevent the alteration of collagen stable isotope compositions by mineral acid demineralization (Carrasco et al., 2018; Tatsch, Secchi & Botta, 2016; Turner Tomaszewicz et al., 2016; Turner Tomaszewicz et al., 2017; Turner Tomaszewicz et al., 2015). Due to the persisting idea that demineralization alters the *δ*^15^N values of bone collagen, other authors have opted to divide each sample into a whole bone fraction for *δ*^15^N analysis and a pre-treated collagen fraction for *δ*^13^C analysis (Avens et al., 2013; Bas et al., 2019a; Ramirez et al., 2015). Not only does this methodology increase the cost of analyses dramatically, but in our opinion, it has not been sufficiently justified based on current experimental data. This methodology also has the potential to confound the results when comparing the isotopic composition of whole bone samples and collagen samples and considering them equivalent regardless of their differing macromolecular components.

Other studies have compared the stable isotope composition of modern and ancient bone collagen treated with two different demineralization agents (EDTA and HCl) and found no significant differences (Tuross, 2012; Tuross, Fogel & Hare, 1988). It should be noted, however, that thus far no studies have performed a quantitative assessment of the differences in elemental and isotopic composition of paired samples left untreated (whole bone) against EDTA-treated samples. Additionally, previous research has not incorporated robust sample sizes of modern and ancient specimens (Carrasco et al., 2018; Tatsch, Secchi & Botta, 2016; Tuross, 2012), leaving a gap in the literature that necessitates a methodological study such as this one to test these hypotheses on a larger number of samples. Furthermore, we compared the elemental composition of collagen samples determined by elemental analysis-isotope ratio mass spectrometry with the elemental compositions determined using amino acid data; to the best of our knowledge, no similar published comparisons exist.

Our research will examine if demineralization using strong acid does indeed alter the stable isotope composition of bone collagen by comparing the *δ*^13^C and *δ*^15^N values of samples treated with HCl and samples treated with the neutral chelating agent EDTA, that preserves collagen structure (Chen et al., 2005; Pang et al., 2021). Amino acid composition data from a subset of the total samples treated with EDTA and HCl was also assessed to help characterize any potential differences in bulk amino acid composition between samples treated with different demineralization agents. We also compared the stable isotope composition of collagen samples with equivalent whole bone samples. If mineral acid demineralization alters collagen *δ*^15^N values, whole bone and EDTA-treated collagen should be characterized by the same isotopic compositions, while HCl-treated collagen should have significantly different *δ*_15_N values relative to both EDTA-treated collagen and whole bone. This experimental design allows us to unambiguously test the effects of demineralization pre-treatment on sample stable isotope composition by eliminating the need to assume that whole bone samples are homogenous and predictable in their chemical composition (Bas & Cardona, 2018; Bas et al., 2019b; Carrasco et al., 2018; Tatsch, Secchi & Botta, 2016; Turner Tomaszewicz et al., 2015).

A secondary objective of this research was to examine the efficacy of untreated whole bone samples as replacements for bone collagen in stable isotope studies. As discussed above, multiple publications have advocated for the use of whole bone samples for stable isotope analysis rather than collagen to eliminate the perceived impact that demineralization has on the stable isotope composition of the sample (Bas et al., 2019a; Carrasco et al., 2018; Tatsch, Secchi & Botta, 2016; Turner Tomaszewicz et al., 2015). We have compared the stable isotope and elemental composition of bone collagen treated with two different agents relative to untreated whole bone to quantify the differences between these treatments. To control for the impact of lipid contaminants, which may be present in the untreated whole bone samples but removed in collagen extracts, we incorporated a treatment that solely involves lipid extraction (LE) to examine the effects that this process may have on sample stable isotope composition.

## Materials & Methods

### Materials

Modern bone samples (*n* = 30) were taken from cow (*Bos taurus*, *n* = 11), muskox (*Ovibos moschatus*, *n* = 1), kangaroo (Macropodidae *spp.*, *n* = 17), and pig (*Sus scrofa*, *n* = 1). The modern bone samples were purchased from an abattoir and consisted of various skeletal elements (Table S1). The ancient samples consisted of the midshaft of ringed seal (*Pusa hispida, n* = 30) fibulae, which were collected from an archaeological site on Somerset Island in the Canadian Arctic (Hazard Inlet, PaJs-13, Field Permit (91-016) granted by the Northwest Territories Government). The site from which these samples were collected is approximately 650 years old (Savelle & Habu, 2004). The ancient samples chosen for this analysis were well preserved and lacked dark coloration to reduce the likelihood of contamination with humic acids from the burial environment (Schnitzer & Khan, 1975; Szpak, Krippner & Richards, 2017).

### Bone pre-treatment

**Figure 1:**
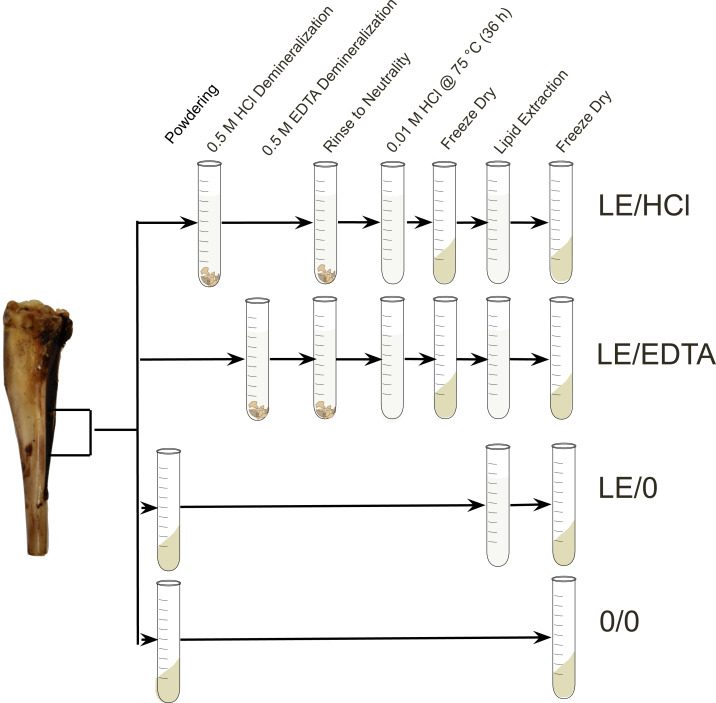
Experimental design. Diagram depicting the steps involved in each experimental method (Szpak, Krippner & Richards, 2017).

Bone samples were cleaned of any adhering surface material using an Ultimate XL-D micromotor with a diamond-tipped cutting wheel (NSK-Nakanishi International, Kanuma, Tochigi, Japan). A single aliquot of cortical bone was cut from each sample and placed in a Plattner mortar to homogenize the sample. The crushed samples were then funneled through a metal sieve to isolate a 1–2 mm size fraction (bone chunks) to be used for the demineralization treatments and a<0.18 mm size fraction (bone powder) to be used for the whole bone samples. The samples destined for pre-treatment were then divided into different culture tubes and processed using the various methodologies (Fig. 1). For a subset of the modern samples, replicate samples were prepared and analyzed to assess the difference in homogeneity between whole bone and collagen (Fig. S1, Table S2).

For the LE/HCl treatment, 200–300 mg of bone was immersed in 0.5 M HCl, and the solution was changed every 24 h until the samples appeared visually demineralized. The samples were left on an orbital shaker between solution changes to increase the rate of demineralization and ensure the bone fragments demineralized evenly. The samples were demineralized at room temperature. Post-treatment, the samples were rinsed in Type I water (resistivity >18.2 M Ω⋅ cm) three times and then solubilized in 0.01 M HCl at 75 °C for 36 h. Following solubilization, the samples were lyophilized and then lipids were removed *via* a phase separation. Lipid removal was performed using 6 mL of 2:1 chloroform:methanol added to 1.6 mL of Type I water containing the dissolved ‘collagen’ (Folch, Lees & Stanley, 1957). The lipid extraction was performed three times and the soluble collagen was then lyophilized.

The LE/EDTA treatment used the protocol described above except for the demineralization process. For the EDTA demineralized samples, 200–300 mg of bone was placed in 5 mL of 0.5 M EDTA solution at a pH of 7.4 (Koon, 2012; Simpson et al., 2016). The solution was changed every 3 days and samples were left on an orbital shaker in between solution changes (Simpson et al., 2016). Prior to refluxation, the samples were rinsed 15 times with Type I water to ensure that any residual EDTA was removed (Tuross, Fogel & Hare, 1988). For the LE/0 treatment, 50–200 mg of bone powder was rinsed in 6 mL of 2:1 chloroform:methanol three times in order to remove lipids (Folch, Lees & Stanley, 1957). Following lipid extraction, the bone powder was lyophilized to ensure that water was removed from the sample prior to analysis. The final 0/0 treatment used whole bone powder left untreated. After the samples from each treatment were lyophilized, 0.45−0.55 mg of each sample was weighed into a tin capsule for analysis.

### Stable isotope analysis and quality control

An elemental analyzer (EA) coupled to a continuous flow isotope ratio mass spectrometer (CF-IRMS) was used to determine the isotopic and elemental compositions of the samples. All modern samples were analyzed using a Thermo Finnigan Delta V CF-IRMS coupled to a Costech 4010 EA at the Laboratory for Stable Isotope Science (London, ON, Canada). The ancient samples were analyzed using a Nu Horizon CF-IRMS coupled to a Euro EA 3000 at the Trent University Water Quality Centre (Peterborough, ON, Canada). Full details on the standard reference materials used for calibration and monitoring accuracy are presented in the supplemental information (Tables S3, S4, S5, S6). The full elemental and isotopic results for all samples are also presented in the supplemental information (Table S7).

### Amino acid analysis

To assess the difference in amino acid composition of collagen samples treated with different chemical agents (EDTA/0 and HCl/0), a subset of the modern (*n* = 6) and ancient (*n* = 6) samples were analyzed by Acquity ultra performance liquid chromatography (UPLC). The analyses were performed by the SPARC BioCenter at Sick Kids Hospital, (Toronto, ON, Canada). The six samples that had the largest difference in atomic C:N ratio in EDTA-treated and HCl-treated samples from the modern and ancient contexts were selected. The amino acid composition of the collagen samples was compared qualitatively between the different treatments (EDTA/0 and HCl/0). Using the counts of amino acid residues per 1000 residues, the atomic C:N ratios of the samples were calculated based on the expected number of carbon and nitrogen atoms in each residue.

### Statistical analysis

All statistical tests were conducted using PAST version 4.05 (Hammer, Harper & Ryan, 2001). The isotopic composition of paired samples grouped by demineralization agent were compared using a Wilcoxon test. The distribution of sample isotopic composition grouped by treatment was not normal (based on Shapiro Wilks tests), which is expected given that the samples were from a variety of different species with highly variable feeding ecologies. The elemental composition was compared between treatments using a Mann Whitney U test for non-paired samples.

## Results

### Isotopic compositions of modern samples

**Figure 2:**
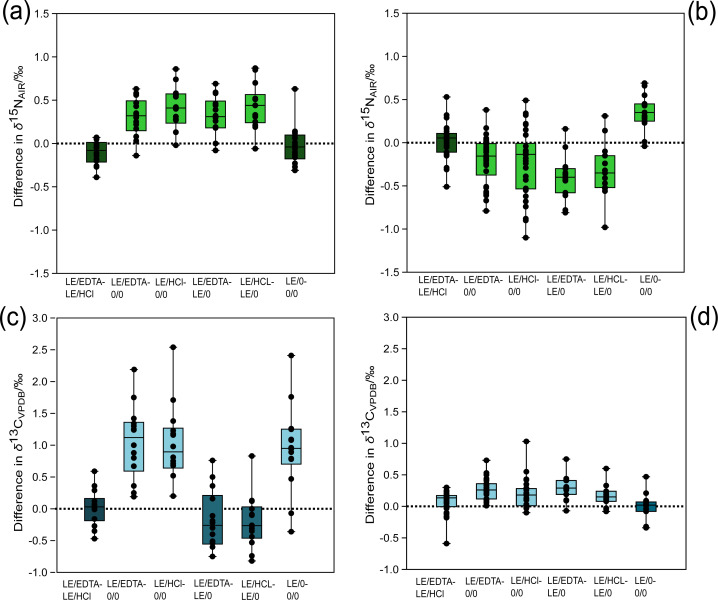
Difference in stable isotope composition between treatments for modern and ancient samples. (A) Modern *δ*^15^N comparison, (B) ancient *δ*^15^N comparison, (C) modern *δ*^13^C comparison, (D) ancient *δ*^13^C comparison. The light-colored boxes represent comparisons associated with statistically significant differences (*p* values in Tables 2 and 3). The dashed horizontal line in each panel denotes no isotopic difference between the treatments being compared. Points falling above the line demonstrate that the first treatment has a higher (less negative) *δ*^13^C or *δ*^15^N value than the second treatment and vice versa if the point plots below the line.

Regardless of which demineralization agent was used (EDTA or HCl), the isotopic compositions of collagen samples were not significantly different (Tables 2 and 3). The average differences in the *δ*^13^C and *δ*^15^N values between the two demineralization treatments were both 0.04 ‰ (Fig. 2). Comparisons between either of the demineralization treatments (LE/HCl and LE/EDTA) and the whole bone treatment (0/0) yielded statistically significant differences for *δ*^13^C and *δ*^15^N (Tables 2 and 3). In general, the *δ*^13^C and *δ*^15^N values were higher in demineralized samples as compared to whole bone samples (0/0) (Fig. 2). Among the demineralization treatments and the lipid-extracted whole bone treatment (LE/0), there were significant differences in the *δ*^15^N values, but not in the *δ*^13^C values (Tables 2 and 3). The *δ*^13^C values had an average difference of 0.20‰ when comparing HCl-treated and lipid-extracted whole bone, and an average difference of 0.19‰ when comparing EDTA-treated samples and lipid-extracted whole bone (Fig. 2). Additionally, the *δ*^15^N values were not significantly different for whole bone samples before and after lipid extraction (Table 2). The *δ*^13^C values were, however, significantly different, with the values being higher on average in the whole bone samples post-lipid extraction (Table 3).

**Table 2:** *p* values for Wilcoxon tests comparing *δ*^15^N between treatments. Bolded values represent comparisons yielding *p* values below the alpha threshold (0.05).

Samples	df	Treatment	LE/HCl	LE/EDTA	LE/0
Modern	48	LE/EDTA LE/0 0/0	0.26 **0.001** **<0.001**	**—** **0.001** **0.001**	**—** **—** 0.43
Ancient	29	LE/EDTA LE/0 0/0	0.33 **0.003** **0.007**	— **<0.001** **<0.001**	— — **<0.001**

**Table 3:** *p* values for Wilcoxon tests comparing *δ*^13^C between treatments. Bolded values represent comparisons yielding *p* values below the alpha threshold (0.05).

Samples	df	Treatment	LE/HCl	LE/EDTA	LE/0
Modern	48	LE/EDTA LE/0 0/0	0.32 0.07 **<0.001**	**—** 0.16 **<0.001**	**—** **—** **<0.001**
Ancient	29	LE/EDTA LE/0 0/0	**0.008** **0.002** **<0.001**	— **0.002** **<0.001**	— — 0.73

**Figure 3:**
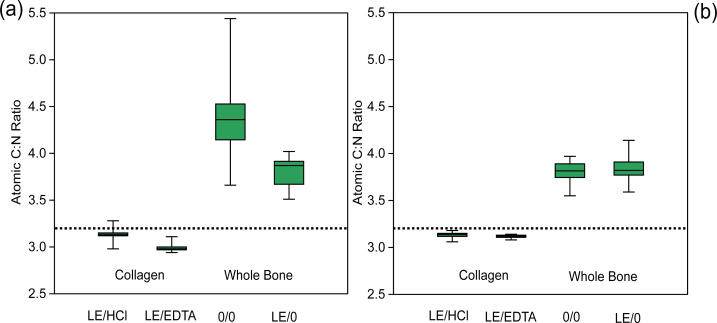
Atomic C:N ratio is higher in whole bone samples. Comparison of atomic C:N ratios among various treatments. (A) Modern sample atomic C:N ratios, (B) ancient sample atomic C:N ratios. The dotted line represents the average expected atomic C:N ratio of modern mammalian collagen (3.22) (Guiry & Szpak, 2020).

### Isotopic compositions of ancient samples

For the ancient samples, there were no significant differences in the *δ*^15^N values between demineralization pre-treatments (LE/HCl *vs*. LE/EDTA). There were, however, significant differences in the *δ*^13^C values between the demineralized samples (LE/HCl *vs.* LE/EDTA) (Table 3). The average difference in *δ*^13^C values between LE/EDTA and LE/HCl treated samples was 0.07‰ (Fig. 2). Among the demineralized treatments and the whole bone treatment (0/0), there were significant differences in both the *δ*^13^C and *δ*^15^N values (Tables 2 and 3). The average difference in *δ*^13^C between HCl-treated and whole bone samples was 0.19‰, while the average difference in *δ*^15^N was 0.23‰ (Fig. 2). When comparing the lipid-extracted whole bone (LE/0) and either of the demineralization treatments, the *δ*^13^C and *δ*^15^N values were significantly different (Tables 2 and 3). For the comparison of lipid-extracted whole bone (LE/0) and untreated whole bone (0/0), there were significant differences in the *δ*^15^N values, but not in the *δ*^13^C values (Tables 2 and 3).

### Elemental composition of modern samples

The demineralized samples had significantly different atomic C:N ratios depending on the demineralization agent used (EDTA or HCl) (Table 4, Fig. 3). On average, the atomic C:N ratio was lower by 0.15 for EDTA-treated samples, due to a proportionally higher wt% N composition as compared to HCl-treated samples. The atomic C:N ratios of collagen samples and whole bone samples were also significantly different for all comparisons, with whole bone samples having consistently higher atomic C:N ratios (Fig. 3). The atomic C:N ratios of lipid-extracted whole bone and untreated whole bone were significantly different in the modern samples, with higher C:N ratios observed in the untreated whole bone (Fig. 3).

**Table 4:** *p* value results for Mann Whitney U tests comparing atomic C:N ratio between treatments. Bolded values represent comparisons yielding *p* values below the alpha threshold (0.05).

Samples	df	Treatment	LE/HCl	LE/EDTA	LE/0
Modern	48	LE/EDTA LE/0 0/0	**<0.001** **<0.001** **<0.001**	**—** **<0.001** **<0.001**	**—** **—** **<0.001**
Ancient	29	LE/EDTA LE/0 0/0	**0.001** **<0.001** **<0.001**	— **<0.001** **<0.001**	— — 0.92

### Elemental composition of ancient samples

**Figure 4:**
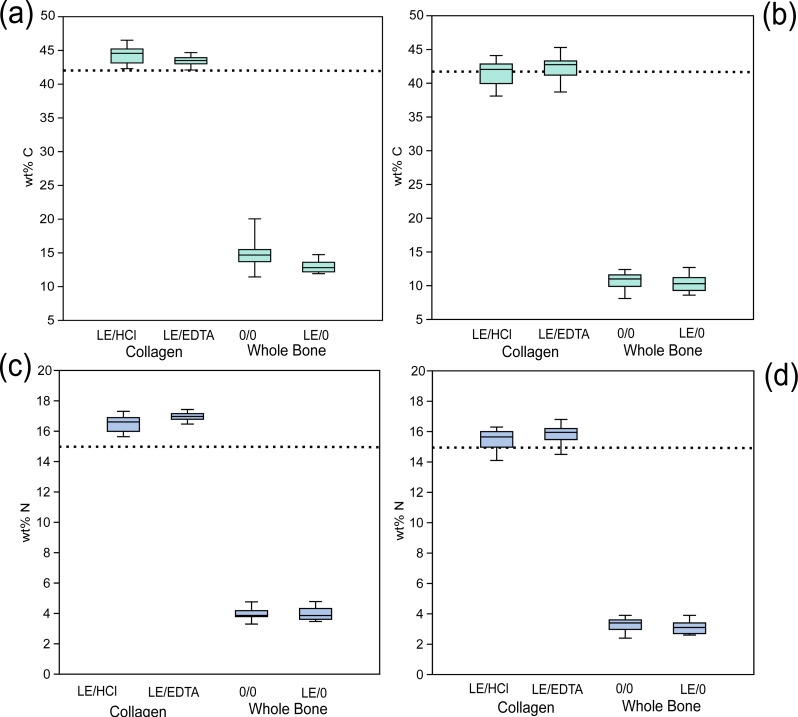
Whole bone samples have lower wt %C and wt % N than collagen. Comparison of elemental composition of various treatments. (A) Modern wt% C, (B) ancient wt% C, (C) modern wt% N, (D) ancient wt% N. The dotted line represents the average expected wt% C and wt% N of modern mammalian collagen (42.4% and 15.3% respectively) (Guiry & Szpak, 2020).

In the ancient context, collagen samples had significantly different atomic C:N ratios depending on the demineralization agent used (EDTA or HCl) (Table 4, Fig. 3). The average atomic C:N ratios for ancient collagen samples treated with EDTA and HCl were, however, quite similar (3.12 ± 0.02 and 3.13 ± 0.03 respectively) (Table 4, Fig. 3). The wt% N values were lower on average in HCl-treated samples as compared to EDTA-treated samples for ancient specimens (Fig. 4). Any comparisons between a collagen sample (LE/EDTA and LE/HCl) and a whole bone sample (0/0 and LE/0) also yielded statistically different atomic C:N ratios (Table 4, Fig. 3). Both the wt% C and wt% N values were higher on average in the collagen samples than the whole bone samples (Tables S8, S9, S10, Fig. 4). The atomic C:N ratios of lipid-extracted whole bone and untreated whole bone were not significantly different in the ancient samples (Table 4). Collagen yield was significantly higher (*p* < 0.001) in HCl-treated samples than EDTA-treated samples (Table S11).

### Amino acid composition data

The amino acid composition of samples treated with EDTA and HCl are characteristic of collagenous proteins, with approximately 1/3 of the amino acids being glycine, and a relatively high proportion of proline and hydroxyproline relative to the other amino acids (Tables S12, S13). A qualitative assessment of paired samples treated with EDTA and HCl shows no systematic differences in the proportion or total amount of amino acids (Tables S12, S13). The atomic C:N ratios of samples treated with HCl as measured by EA-IRMS and determined using amino acid composition data are quite different, while the atomic C:N ratios determined by both methods are similar for samples treated with EDTA (Table S14). The atomic C:N ratio for all samples treated with HCl as calculated using amino acid data are systematically lower than the C:N ratios measured by EA-IRMS (Table S14).

## Discussion

### Effect of acidification on collagen stable isotope composition

Based on the stable isotope compositions of samples treated with HCl and EDTA, there is no evidence that demineralization using a strong acid alters the stable isotope composition of bone collagen. In both modern and ancient samples, the *δ*^13^C and *δ*^15^N values of bone collagen were not statistically different depending on which demineralization agent was used. The exception to this is the *δ*^13^C values of HCl and EDTA-treated ancient samples, however, the average difference between treatments was very low (0.07 ‰). The magnitude of this difference is less than the analytical uncertainty of these analyses and therefore it would not have a significant impact on any interpretations generated using these data. Interpretations should only be made for comparisons yielding differences beyond the analytical uncertainty of the analyses. HCl is an effective chemical agent for the demineralization of bone to be used in stable isotope research, given that it does not alter the stable isotope composition of bone collagen and the isolated material has amino acid and elemental compositions closely matching those expected for collagenous proteins. The results from these comparisons are consistent with previous findings that EDTA-treated, and HCl-treated bone collagen have similar stable isotope compositions (Tuross, 2012; Tuross, Fogel & Hare, 1988). These results contradict previous assertions that HCl alters the stable isotope composition of bone collagen (Bas & Cardona, 2018; Bas et al., 2019b; Turner Tomaszewicz et al., 2015).

Previous studies that have made claims about the impact of HCl on collagen isotopic compositions compared HCl-demineralized collagen extracts against whole bone samples that were left untreated (Bas et al., 2019b). These studies found that either the *δ*^13^C and/or *δ*^15^N values were significantly different between these treatments (Bas et al., 2019b; Tatsch, Secchi & Botta, 2016; Turner Tomaszewicz et al., 2015). Based on these data, the discrepancy in stable isotope composition between collagen and whole bone has been interpreted to be due to the effects of HCl demineralization. Our results contradict this hypothesis because demineralization performed using a neutral agent (EDTA) and mineral acid (HCl) produced very similar isotopic compositions (Fig. 2), indicating that acidification is not the cause of the differing stable isotope compositions observed in these other studies.

### Stable isotope and elemental composition of whole bone *versus* collagen

The stable isotope data comparing the different sample materials suggests that whole bone samples should not be analyzed in the place of purified bone collagen. In both modern and ancient contexts, the whole bone samples had significantly different *δ*^13^C and *δ*^15^N values as compared to collagen samples. The sole exception to this is the comparison between the *δ*^13^C values of modern lipid-extracted whole bone and collagen extracts, which did not yield a significant difference (Table 3). Moreover, while the collagen samples had elemental compositions that approximate the expected average atomic C:N ratio for modern mammalian collagen (Guiry & Szpak, 2020), the atomic C:N ratios of whole bone samples were significantly higher and more variable (Fig. 3, Table S8). These results contradict previous assertions supporting the use of whole bone as a useful analytical substrate (Bas et al., 2019a; Carrasco et al., 2018; Turner Tomaszewicz et al., 2015).

### *δ*^13^C values

For the modern samples, the *δ*^13^C values of bone collagen were *higher* on average than those of the whole bone samples (0/0) (Fig. 2). These results suggest that the whole bone samples contained lipids with low *δ*^13^C (DeNiro & Epstein, 1977), which skewed the *δ*^13^C values lower relative to collagen samples. By contrast the modern collagen samples had *lower δ*^13^C values on average relative to the lipid-extracted (LE/0) whole bone samples, suggesting that the presence of high *δ*^13^C bioapatite had a greater effect on the isotopic composition of the whole bone samples after lipids were removed (Sullivan & Krueger, 1981). These data provide evidence that the presence of high *δ*^13^C bioapatite *and* low *δ*^13^C lipids can affect the sample stable isotope composition of whole bone relative to bone collagen. In the ancient samples the *δ*^13^C values of collagen were consistently higher than both the untreated whole bone, and the lipid-extracted whole bone. It is unlikely that lipids were preserved in the ancient samples, based on the lack of significant differences between the LE/0 and 0/0 *δ*^13^C values and atomic C:N ratios (Tables 3 and 4). Had lipids been present, we would expect to see the *δ*^13^C values increase significantly post-lipid extraction (DeNiro & Epstein, 1977), while the atomic C:N ratios would decrease, such as is observed in the modern samples. These trends were not observed in the ancient samples (Fig. 2). Based on the evidence supporting the lack of lipid contaminants, there is likely another contaminant responsible for the trend observed in the ancient samples. Low *δ*^13^C fulvic acids derived from the burial environment may be responsible for the signal observed in the whole bone samples (Webb, Schwarcz & Healy, 2004). Fulvic acids are soluble in acidic solution (Lobartini, Orioli & Tan, 1997), meaning they are unlikely to be present in the HCl-treated collagen samples. It is possible that low *δ*^13^C fulvic acids in the whole bone samples skewed the stable isotope composition of the whole bone samples (0/0 and LE/0) lower relative to the collagen samples.

### *δ*^15^N values

Despite the fact that EDTA-treated and HCl-treated collagen samples had very similar *δ*^15^N values, the *δ*^15^N values of collagen extracts and whole bone samples were significantly different. The modern collagen samples had consistently higher *δ*^15^N values as compared to both whole bone treatments (LE/0 and 0/0). These results may be due to the presence of non-collagenous proteins in the whole bone samples that are composed of different proportions of source (essential) and trophic (non-essential) amino acids as compared to the collagen (Gundberg et al., 1984). Given that most non-collagenous proteins are tightly bound to the mineral matrix (Gundberg et al., 1984), it is feasible that they are at least partially removed during the demineralization process (Termine et al., 1981). If the non-collagenous proteins are, however, retained in the whole bone samples, they may lower the *δ*^15^N values of the whole bone relative to the collagen. The higher atomic C:N ratios in the whole bone samples as compared to the collagen samples are also consistent with our suggestion that a substantial amount of non-collagenous proteins, which have atomic C:N ratios that are higher than collagen (Guiry & Szpak, 2020), are retained in the whole bone samples. This is particularly problematic since the amount of non-collagenous proteins may vary among different parts of the skeleton and within bones (Roach, 1994). When comparing *δ*^15^N values among different taxa or even skeletal elements, the use of whole bone will unnecessarily increase random error in the measurements.

The ancient samples display the opposite trend in *δ*^15^N values as compared to the modern samples, meaning that *δ*^15^N values are consistently lower in collagen relative to whole bone samples. Given that the isotopic composition of non-collagenous proteins has yet to be characterized, it is also possible that they may be present in the ancient samples (Cappellini et al., 2012). The elemental data supports this hypothesis given the elevated C:N ratios observed in whole bone samples (Guiry & Szpak, 2020). It is also possible, however, that another contaminant from the burial environment has affected the *δ*^15^N values of the ancient samples that were left untreated.

### Elemental compositions

The elemental composition of whole bone samples was significantly different as compared to collagen samples. The wt% C and wt% N of whole bone samples were consistently and significantly lower than would be expected for modern mammalian collagen (Guiry & Szpak, 2020) (Fig. 4, Table S8) likely because much of the sample was composed of inorganic material that was either not combustible or combusted poorly during EA-IRMS analysis and contained relatively little carbon or nitrogen. More importantly, however, the atomic C:N ratios were significantly higher in whole bone samples (4.35 ± 0.53 for modern and 3.81 ± 0.10 for ancient untreated whole bone) and these were characterized by a large amount of variation (Fig. 3). Because these atomic C:N ratios are far higher than what would be expected for pure collagen, a substantial amount of non-collagenous material must also have been combusted. These atomic C:N ratios would largely fail the most commonly-used quality control criteria for both modern (Guiry & Szpak, 2020) and ancient samples (Ambrose, 1990; DeNiro, 1985; Guiry & Szpak, 2021) regardless of whether lipid extraction was performed. In isotope ratio mass spectrometry, one should try to isolate as pure a substance as possible to avoid issues created by heterogeneous samples composed of multiple matrices with variable elemental and isotopic compositions such as bone (Zioupos, Currey & Casinos, 2000). On the basis of this criterion, whole bone makes for a poor analytical substrate and taken as a whole, our data suggest that whole bone samples cannot be used in place of extracted bone collagen. The isotopic and elemental compositions of these two materials are not directly comparable.

### Effect of demineralization on collagen elemental composition

Despite the lack of significant differences in the stable isotope compositions of samples treated with EDTA or HCl, there was a significant difference in the atomic C:N ratio for both modern and ancient samples. On average, samples treated with EDTA had lower atomic C:N ratios, due to a higher wt% N, as compared to the samples treated with HCl. A slight reduction in nitrogen in samples treated with HCl has been observed in a previous methodological study comparing the use of EDTA and HCl for decalcification (Tuross, 2012). The lower atomic C:N ratio in EDTA-treated samples was observed consistently across modern and ancient samples but did not have a significant impact on the stable isotope compositions of the samples.

**Figure 5:**
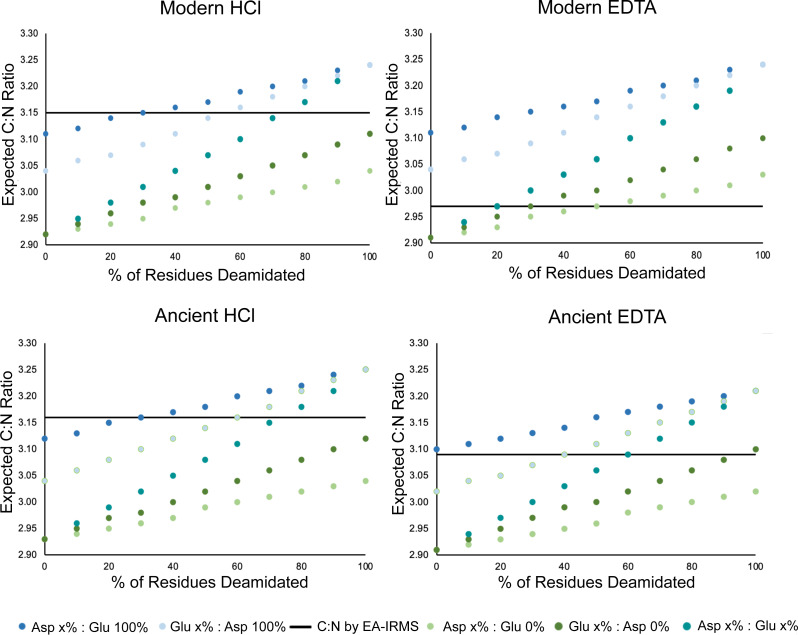
Deamidation rate is higher in HCl-treated samples. Scatterplot comparing the effect of percent glutamine and asparagine deamidation on atomic C:N ratio calculated using amino acid data. The data for each of the four categories was generated by averaging the amino acid compositions of three samples in each category whose amino acid compositions were determined. The black line on each plot represents the average C:N ratio of the three samples as determined by EA-IRMS. The different colors distinguish between amount of deamidation, where x% is the amount of deamidation for either glutamine or asparagine presented on the x axis while the % deamidation of the accompanying amino acid is held constant.

There are multiple mechanisms that may contribute to this observed difference in atomic C:N ratio and wt% C and N composition for EDTA-treated samples. One potential cause for this observed difference is amino acid deamidation (in glutamine and asparagine residues) occurring at a relatively higher rate in the HCl-treated samples, resulting in a disproportionate loss of nitrogen relative to carbon (Cleland, Sarancha & France, 2021; Jacob et al., 2018; Procopio & Buckley, 2017; Simpson et al., 2016). Deamidation of glutamine and asparagine occurs *in vivo*, in the burial environment, and during select pre-treatment procedures (Creecy et al., 2021; Procopio & Buckley, 2017; Schroeter & Cleland, 2016). Glutamine deamidation in bone collagen during demineralization occurs more frequently with a strong acid agent (HCl) as compared to a neutral agent (EDTA) (Procopio & Buckley, 2017; Simpson et al., 2016). Variable rates of deamidation in the EDTA and HCl-treated samples did not result in any discrepancy between the stable isotope composition of the samples. This suggests that deamidation modifications are inconsequential in the context of nitrogen isotope analyses of bulk bone collagen by EA-IRMS. Using the count of amino acid residues per 1,000 residues in the samples, we calculated the expected atomic C:N ratios of the samples based on the number of carbon and nitrogen atoms in each residue assuming no deamidation had occurred in glutamine or asparagine (Table S14). These atomic C:N ratios calculated using amino acid data were systematically lower than the atomic C:N ratios measured by EA-IRMS for HCl treated samples (Table S14). These data suggest that glutamine and/or asparagine deamidation is occurring more frequently in HCl-treated samples, resulting in a relatively greater loss of nitrogen and therefore a higher C:N ratio as measured by EA-IRMS. Our data also suggest that deamidation is occurring in the EDTA-treated samples, although to a lesser extent (Fig. 5). We modeled the amount of deamidation occurring in the EDTA and HCl treated samples to generate an estimate of the relative rates of deamidation among treatments and for modern and ancient samples. The expected rate of deamidation was consistently higher in HCl samples than in EDTA samples (Fig. 5).

We can exclude the possibility that EDTA (a nitrogen and carbon containing compound) has contaminated the EDTA-treated samples. The EDTA used for this experiment had *δ*^13^C values around –38.00 ‰ and *δ*^15^N values around + 0.15 ‰ (both of which are much lower than the *δ*^13^C and *δ*^15^N values of any of the collagen extracts being analyzed). If EDTA contamination was present, it would skew the stable isotope composition of EDTA-treated samples relative to the HCl-treated samples, which was not observed in our results. Contamination with EDTA would also cause higher atomic C:N ratios, given that this compound has an atomic C:N ratio of 5. Although EDTA treatment did not affect the stable isotope composition of bone collagen, we have not tested the impact of this treatment on radiocarbon dating. We caution future researchers who may choose to employ EDTA demineralization in the preparation of bone samples for radiocarbon dating to further investigate the possibility of carbon contamination from EDTA treatment.

Despite the differences in elemental composition that are observed, the stable isotope composition of EDTA-treated and HCl-treated samples are not significantly different. As a result, these differences in elemental composition are of no concern for stable isotope studies relying on collagen as a sample material. These data do, however, suggest that various quality control criteria for bone collagen derived from EA-IRMS elemental data do not apply equally well to collagen extracted with EDTA and HCl (Ambrose, 1990; DeNiro, 1985; Guiry & Szpak, 2020; Guiry & Szpak, 2021). Since EDTA-treated collagen produces much lower atomic C:N ratios when measured by EA-IRMS than HCl-treated collagen, significant lipid contamination (for modern samples) or humic contamination (for ancient samples) would occur at a lower atomic C:N ratio than the traditionally-used cutoff point of 3.60 (DeNiro, 1985). Those using EDTA as a demineralization agent should work towards developing their own set of quality control criteria, particularly as it relates to atomic C:N ratios rather than relying on elemental quality control criteria that were developed or tested using HCl as a demineralization agent.

We investigated the impact of demineralization agent on the bulk stable isotope composition of bone collagen. The detailed mechanisms underlying the demineralization treatments should be explored in future studies using techniques such as MS/MS to further characterize the impact of demineralization agent and the effect of demineralization time on modern and ancient proteomes. Additionally, our analyses were limited to modern and well-preserved ancient samples, thus the field of research would benefit from further testing of these demineralization techniques on poorly preserved bone samples.

## Conclusion

• HCl demineralization does not alter the stable isotope composition of bone collagen to a significant degree.
• These data indicate that both HCl and EDTA demineralization can be used to produce collagen samples with accurate and mutually comparable stable isotope compositions.
• Whole bone samples are not effective replacements for collagen samples given that the stable isotope composition of whole bone samples are significantly different than those of collagen extracted using two different methods.
• In future studies examining the stable isotope composition of bone collagen, pre-treatment *via* demineralization should be conducted to obtain reliable and accurate data.
• Methodological studies aiming to assess the influence of various pre-treatments on the isotopic compositions of bone collagen should not make comparisons relative to whole bone as this material is heterogeneous in terms of its elemental and isotopic composition, making it a poor analytical substrate for isotopic analysis.

##  Supplemental Information

10.7717/peerj.13593/supp-1 Supplemental Information 1 Supplemental Information.

10.7717/peerj.13593/supp-2 Supplemental Information 2 Replicate samples had similar stable isotope compositions within treatments Boxplots depicting the difference in (A) *δ*^15^N, (B) *δ*^13^C values among replicates of the same samples. Differences between each of the replicates of the same samples were then pooled with all five samples from the same treatment. The dotted line represents a difference of zero.

## Additional Information and Declarations

### Field Study Permissions

The following information was supplied relating to field study approvals (i.e., approving body and any reference numbers): Northwest Territories Government 91-016.

### Data Availability

The following information was supplied regarding data availability: The isotopic data is available in the Supplementary File.
