# Supplementary material for: Acidification does not alter the stable isotope composition of bone collagen
Source: PeerJ. 2022 Jun 14;10:e13593. doi: 10.7717/peerj.13593 (PMC9205305; doi:10.7717/peerj.13593)
Supplement: Supplemental Information 1 [file peerj-10-13593-s001.pdf]

Supplemental Information

Sample Information

In the following table, the species, skeletal element, and anatomical site for the specimens used in this research are presented.

Table S1. Skeletal element and anatomical site sampled for stable isotope and elemental analysis

Sample ID	Species	Context	Skeletal Element	Anatomical Site
11500	Muskox	Modern	**Mandible**	**Body**
11501	Cow	Modern	**Radius**	**Midshaft**
11502	Kangaroo	Modern	**Tibia**	**Distal**
11503	Kangaroo	Modern	**Tibia**	**Distal**
11504	Kangaroo	Modern	**Tibia**	**Distal**
11506	Kangaroo	Modern	**Tibia**	**Distal**
11507	Kangaroo	Modern	**Tibia**	**Distal**
11508	Kangaroo	Modern	**Tibia**	**Distal**
11509	Kangaroo	Modern	**Tibia**	**Distal**
11510	Kangaroo	Modern	**Tibia**	**Distal**
11511	Kangaroo	Modern	**Tibia**	**Distal**
11512	Kangaroo	Modern	**Tibia**	**Distal**
11513	Kangaroo	Modern	**Tibia**	**Distal**
11514	Pig	Modern	**Unknown**	**Unknown**
11515	Cow	Modern	**Radius**	**Midshaft**
11516	Cow	Modern	**Radius**	**Midshaft**
11517	Cow	Modern	**Radius**	**Midshaft**
11518	Cow	Modern	**Tibia**	**Distal**
10788	Kangaroo	Modern	**Tibia**	**Distal**
10787	Kangaroo	Modern	**Tibia**	**Distal**
10786	Kangaroo	Modern	**Tibia**	**Distal**
10785	Kangaroo	Modern	**Tibia**	**Distal**
10784	Kangaroo	Modern	**Tibia**	**Distal**
10263	Cow	Modern	**Radius**	**Midshaft**
10247	Cow	Modern	**Tibia**	**Distal**
3619	Cow	Modern	**Tibia**	**Distal**
1284	Cow	Modern	**Tibia**	**Distal**
1260	Cow	Modern	**Tibia**	**Distal**
12092	Cow	Modern	**Tibia**	**Distal**
13003	Ringed Seal	Ancient	**Fibula**	**Midshaft**
13005	Ringed Seal	Ancient	**Fibula**	**Midshaft**
13008	Ringed Seal	Ancient	**Fibula**	**Midshaft**
13009	Ringed Seal	Ancient	**Fibula**	**Midshaft**
13010	Ringed Seal	Ancient	**Fibula**	**Midshaft**
13012	Ringed Seal	Ancient	**Fibula**	**Midshaft**
13015	Ringed Seal	Ancient	**Fibula**	**Midshaft**
13019	Ringed Seal	Ancient	**Fibula**	**Midshaft**
14080	Ringed Seal	Ancient	**Fibula**	**Midshaft**
14082	Ringed Seal	Ancient	**Fibula**	**Midshaft**
14087	Ringed Seal	Ancient	**Fibula**	**Midshaft**
14089	Ringed Seal	Ancient	**Fibula**	**Midshaft**
14091	Ringed Seal	Ancient	**Fibula**	**Midshaft**
14097	Ringed Seal	Ancient	**Fibula**	**Midshaft**
14105	Ringed Seal	Ancient	**Fibula**	**Midshaft**
14106	Ringed Seal	Ancient	**Fibula**	**Midshaft**
14108	Ringed Seal	Ancient	**Fibula**	**Midshaft**
14112	Ringed Seal	Ancient	**Fibula**	**Midshaft**
14113	Ringed Seal	Ancient	**Fibula**	**Midshaft**

14120	Ringed Seal	Ancient	**Fibula**	**Midshaft**
14121	Ringed Seal	Ancient	**Fibula**	**Midshaft**
14125	Ringed Seal	Ancient	**Fibula**	**Midshaft**
14127	Ringed Seal	Ancient	**Fibula**	**Midshaft**
14131	Ringed Seal	Ancient	**Fibula**	**Midshaft**
14140	Ringed Seal	Ancient	**Fibula**	**Midshaft**
14145	Ringed Seal	Ancient	**Fibula**	**Midshaft**
14146	Ringed Seal	Ancient	**Fibula**	**Midshaft**
14147	Ringed Seal	Ancient	**Fibula**	**Midshaft**
14149	Ringed Seal	Ancient	**Fibula**	**Midshaft**
14151	Ringed Seal	Ancient	**Fibula**	**Midshaft**

Statistical Results of Replicate Samples

For a subset of the modern samples, replicate samples were prepared and analyzed to assess the difference in homogeneity between whole bone and collagen. Replicate samples were homogenized and then divided into separate tubes for processing. Contrary to duplicate samples used as quality control indicators during stable isotope analysis, the replicate samples were processed independently and therefore can be used to assess the amount of variability within samples and between treatments (Figures S1, S2). Replicates of the ancient samples were not prepared given the additional variables such as diagenesis and contamination that would make the analyses more complex.

Table S2. Standard deviation in stable isotope composition of replicate samples for three different treatments. For all tests $n=5$.

Sample Number	$\pm 1\sigma$ for $\delta^{13}C$ (LE/HCl)	$\pm 1\sigma$ for $\delta^{15}N$ (LE/HCl)	$\pm 1\sigma$ for $\delta^{13}C$ (LE/EDTA)	$\pm 1\sigma$ for $\delta^{15}N$ (LE/EDTA)	$\pm 1\sigma$ for $\delta^{13}C$ (0/0)	$\pm 1\sigma$ for $\delta^{15}N$ (0/0)
11512	0.17	0.17	0.21	0.08	0.10	0.10
11513	0.11	0.03	0.19	0.09	0.13	0.07
11514	0.14	0.18	0.09	0.06	0.05	0.06
11515	0.22	0.06	0.11	0.15	0.09	0.17
11516	0.18	0.06	0.33	0.05	0.15	0.07

Standard deviations of the replicate sample stable isotope compositions were variable amongst samples, with no clear pattern for which treatment produced the most homogenous sample (Table S2). These data suggest that whole bone and collagen samples are approximately equally homogeneous in composition (Fig S1, S2).

Demineralization Length

The modern samples were demineralized in HCl for up to seven days, and in EDTA for up to two weeks. The ancient samples were demineralized in HCl for up to 24 hours, and in EDTA for up to nine days.

Stable Isotope Analysis

The carbon and nitrogen isotopic compositions were calibrated relative to Vienna Pee Dee Belemnite (VPDB) and atmospheric nitrogen (AIR), respectively, using a dual-point calibration with standards USGS40 and USGS41a, USGS63, or USGS66 (Schimmelmann et al. 2016) (Tables S3, S4). To monitor accuracy and precision, in-house collagen reference materials SRM-1 (caribou bone collagen), SRM-2 (walrus bone collagen), and SRM-14 (polar bear collagen) were used for collagen samples (Tables S5, S6). For whole bone samples, in-house laboratory reference materials SRM-17 (phenylalanine), SRM-19 (tyrosine), and SRM-14 (polar bear collagen) were used (Table S5, S6). Different quality control standards were used for collagen and whole bone samples in an attempt to matrix-match the samples and standards as much as possible. Duplicate samples were also dispersed throughout the analysis to monitor the precision of the measurements and assess the homogeneity of the samples. The average difference in $\delta^{15}N$ for duplicate collagen samples was ± 0.07 while the average difference was ± 0.04 for $\delta^{13}C$. For

whole bone samples, the average difference was ±0.14 for δ^{15}N and ±0.10 for δ^{13}C. The analytical uncertainty for collagen samples was ±0.10 for δ^{13}C and ±0.29 for δ^{15}N (Szpak et al. 2017). The analytical uncertainty for whole bone samples was ±0.16 for δ^{13}C and ±0.33 for δ^{15}N (Szpak et al. 2017). Quality control criteria (atomic C:N ratio 2.9-3.6 (DeNiro 1985), minimum wt% C and N of 13.8% and 4.8% respectively (Ambrose 1990)) for bone collagen were upheld for all collagen samples that were included in the analyses, and any samples failing to meet these criteria would have been excluded from the analysis. Diagenesis was of limited concern for the ancient samples because they appeared extremely well-preserved and fell within the quality control range expected for ancient bone collagen.

Calibration and Analytical Uncertainty

The stable isotope and elemental compositions of samples and standards were determined using a continuous flow isotope ratio mass spectrometer. The sample measurements were calibrated using VPDB for δ^{13}C values and AIR for δ^{15}N values.

Table S3. Standard reference materials used for the calibration of δ^{13}C relative to VPDB. The observed standard deviation for standards processed at the Laboratory for Stable Isotope Science (Western) and the Water Quality Center (Trent) are also presented.

Standard Name	Sample Material	Accepted $\delta^{13}C_{VPDB}$/‰	Observed (Western) ± 1σ for δ^{13}C	Observed (Trent) ± 1σ for δ^{13}C
USGS40	Glutamic Acid	− 26.39 ± 0.04	± 0.03	± 0.05
USGS41a	Glutamic Acid	+ 37.63 ± 0.05	± 0.05	± 0.06
USGS63	Caffeine	− 1.17 ± 0.04	N/A	± 0.05
USGS66	Glycine	− 0.67 ± 0.03	N/A	± 0.13

Table S4. Standard reference materials used for the calibration of δ^{15}N relative to AIR. The observed standard deviation for standards processed at the Laboratory for Stable Isotope Science (Western) and the Water Quality Center (Trent) are also presented.

Standard Name	Sample Material	Accepted $\delta^{15}N_{AIR}$/‰	Observed (Western) ± 1σ for δ^{15}N	Observed (Trent) ± 1σ for δ^{15}N
USGS40	Glutamic Acid	− 4.52 ± 0.06	± 0.06	± 0.16
USGS41a	Glutamic Acid	+ 47.57 ± 0.11	± 0.21	± 0.32
USGS63	Caffeine	+ 37.83 ± 0.06	N/A	± 0.31
USGS66	Glycine	+ 40.83 ± 0.15	N/A	± 0.13

The standards presented below were used to measure internal accuracy and precision throughout the analytical runs. The values presented below are average results for the standards calibrated relative to VPDB and AIR.

Table S5. Standard reference materials used to monitor accuracy and precision. The observed δ^{13}C values for standards processed at the Laboratory for Stable Isotope Science (Western) and the Water Quality Center (Trent) are also presented.

Standard Name	Sample Material	Mean $\delta^{13}C_{VPDB}$/‰	Observed (Western) $\delta^{13}C_{VPDB}$/‰	Observed (Trent) $\delta^{13}C_{VPDB}$/‰
SRM-1	Caribou bone collagen	− 19.40 ± 0.08	− 19.41 ± 0.06	−19.40 ± 0.07
SRM-2	Walrus bone collagen	− 14.82 ± 0.06	N/A	− 14.81 ± 0.04
SRM-14	Polar bear bone collagen	− 13.68 ± 0.08	− 13.68 ± 0.03	− 13.58 ± 0.11
SRM-17	Phenylalanine	− 12.45 ± 0.04	− 12.48 ± 0.05	− 12.34 ± 0.13
SRM-19	Tyrosine	− 22.97 ± 0.03	− 22.96 ± 0.03	− 22.90 ± 0.07

Table S6. Standard reference materials used to monitor accuracy and precision. The observed δ^{15}N values for standards processed at the Laboratory for Stable Isotope Science (Western) and the Water Quality Center (Trent) are also presented.

Standard Name	Sample Material	Mean $\delta^{15}N_{AIR}$/‰	Observed (Western) $\delta^{15}N_{AIR}$/‰	Observed (Trent) $\delta^{15}N_{AIR}$/‰
SRM-1	Caribou bone collagen	+ 1.82 ± 0.11	+ 1.93 ± 0.09	+ 1.86 ± 0.10
SRM-2	Walrus bone collagen	+ 15.60 ± 0.14	N/A	+ 15.47 ± 0.14
SRM-14	Polar bear bone collagen	+ 21.61 ± 0.16	+ 21.52 ± 0.39	+ 21.70 ± 0.27
SRM-17	Phenylalanine	+ 3.17 ± 0.15	+ 3.65 ± 0.13	+ 3.21 ± 0.24
SRM-19	Tyrosine	+ 7.66 ± 0.15	N/A	+ 7.75 ± 0.04

Isotopic and Elemental Data

The following table contains the entirety of the elemental and isotopic data collected throughout this study, grouped by the treatment type.

Table S7. Elemental and isotopic compositions of samples analyzed, grouped by treatment type

**Bone ID**	**Sample ID**	**Context**	**$\delta^{13}C_{VPDB}$/‰**	**$\delta^{15}N_{AIR}$/‰**	**wt% C**	**wt% N**	**Atomic C:N**	**Collagen Yield**
**LE/EDTA (Collagen)**								
11500	11578	Modern	-22.45	5.53	43.1	16.9	2.98	14.4
11501	11579	Modern	-18.01	5.66	43.5	16.9	3.00	16.5
11502	11580	Modern	-19.05	11.01	43.4	17.2	2.94	15.3
11503	11581	Modern	-19.49	10.47	43.1	17.0	2.96	17.5
11504	11582	Modern	-21.76	11.86	43.7	17.1	2.97	15.4
11506	11584	Modern	-17.2	8.76	43.0	16.9	2.97	16.7
11507	11585	Modern	-16.60	11.72	43.8	17.2	2.97	14.5
11508	11586	Modern	-17.67	8.86	43.7	17.2	2.96	14.6
11509	11587	Modern	-17.87	10.72	42.1	16.5	2.98	13.6
11510	11588	Modern	-18.63	9.32	42.8	16.9	2.95	14.3
11511	11589	Modern	-17.23	11.53	43.0	17.0	2.96	12.0
11512	11590A	Modern	-15.99	11.69	43.5	17.1	2.96	16.1
	11590B	Modern	-16.04	11.73	42.8	16.9	2.95	13.8
	11590C	Modern	-15.77	11.78	43.4	17.1	2.96	13.7
	11590D	Modern	-15.77	11.77	42.7	16.9	2.95	14.1
	11590E	Modern	-16.26	11.57	43.6	17.2	2.96	15.6
11513	11591A	Modern	-18.18	10.73	43.4	17.1	2.97	14.6
	11591B	Modern	-18.65	10.62	44.1	17.3	2.97	15.3
	11591C	Modern	-18.47	10.57	42.7	16.8	2.96	14.7
	11591D	Modern	-18.5	10.52	43.5	17.1	2.97	16.3
	11591E	Modern	-18.62	10.51	43.7	17.1	2.97	16.2
11514	11592A	Modern	-19.96	4.58	43.9	16.6	3.09	13.6
	11592B	Modern	-19.95	4.44	44.0	16.6	3.09	13.5
	11592C	Modern	-19.77	4.54	43.8	16.6	3.09	14.3
	11592D	Modern	-19.94	4.48	44.2	16.7	3.09	13.5
	11592E	Modern	-19.80	4.54	43.9	16.5	3.11	14.6
11515	11593A	Modern	-22.06	5.12	43.3	16.9	2.99	12.4
	11593B	Modern	-22.07	4.89	43.9	17.2	2.98	13.8
	11593C	Modern	-21.96	5.20	43.5	17.1	2.97	13.8
	11593D	Modern	-22.15	5.05	42.2	16.5	2.99	14.4
	11593E	Modern	-21.86	5.27	43.2	16.9	2.99	14.1
11516	11594A	Modern	-22.74	5.85	42.6	16.5	3.00	14.1
	11594B	Modern	-22.10	5.81	43.4	16.9	3.00	13.2

	11594C	Modern	-22.01	5.71	42.7	16.6	3.00	13.7
	11594D	Modern	-21.92	5.78	42.9	16.7	2.99	12.9
	11594E	Modern	-22.05	5.83	43.3	16.8	3.01	13.2
11517	11595	Modern	-14.71	6.25	43.9	17.1	2.99	16.0
11518	11596	Modern	-17.69	6.46	44.7	17.3	3.00	15.6
10788	11597	Modern	-16.61	12.11	44.3	17.4	2.97	15.7
10787	11598	Modern	-17.09	9.76	43.2	16.8	2.99	12.9
10786	11599	Modern	-21.64	9.22	42.5	16.7	2.98	8.7
10785	11600	Modern	-20.78	7.52	44.1	17.3	2.98	13.3
10784	11601	Modern	-15.92	10.97	43.8	17.1	3.00	12.9
10263	11602	Modern	-12.82	7.08	44.6	17.4	2.99	16.2
10247	11603	Modern	-20.95	6.19	44.0	17.0	3.01	16.7
3619	11604†	Modern	-14.67	9.29	89.9	36.0	2.91	15.8
1284	11605	Modern	-23.05	3.56	44.1	17.2	2.99	16.8
1260	11606	Modern	-22.94	3.21	44.7	17.4	2.99	16.2
12092	11607	Modern	-20.92	6.38	44.2	17.2	3.01	15.2
13003	11748	Ancient	-13.61	16.56	39.5	14.9	3.10	7.6
13005	11749	Ancient	-13.83	16.32	41.6	15.5	3.14	11.9
13008	11750	Ancient	-12.85	16.42	42.0	15.7	3.11	11.7
13009	11751	Ancient	-13.76	16.70	41.7	15.8	3.09	7.4
13010	11752	Ancient	-13.27	15.59	40.4	15.2	3.10	6.8
13012	11753	Ancient	-14.57	18.35	41.2	15.5	3.11	8.0
13015	11754	Ancient	-13.42	16.54	41.6	15.8	3.08	7.7
13019	11755	Ancient	-13.47	16.28	41.8	15.9	3.09	7.9
14080	11756	Ancient	-13.39	16.97	42.7	16.2	3.07	13.6
14082	11757	Ancient	-15.08	19.46	42.6	16.2	3.07	12.7
14087	11758	Ancient	-12.87	18.95	43.8	16.7	3.07	15.7
14089	11759	Ancient	-13.56	17.81	43.6	16.7	3.06	13.2
14091	11760	Ancient	-13.93	17.08	42.7	16.4	3.04	12.5
14097	11761	Ancient	-13.93	17.55	43.7	16.2	3.14	15.1
14105	11762	Ancient	-13.01	18.94	43.1	16.0	3.13	10.1
14106	11763	Ancient	-13.86	17.60	43.7	16.3	3.13	9.2
14108	11764	Ancient	-13.81	18.44	43.9	16.3	3.15	11.0
14112	11765	Ancient	-13.73	18.25	43.7	16.2	3.13	13.1
14113	11766	Ancient	-13.64	18.15	43.8	16.4	3.12	11.2
14120	11767	Ancient	-13.73	18.07	43.2	16.3	3.09	12.2
14121	11768	Ancient	-13.42	16.99	43.2	16.3	3.09	8.8
14125	11769	Ancient	-13.83	18.57	42.7	16.0	3.10	11.4
14127	11770	Ancient	-14.12	17.86	43.8	16.4	3.11	12.0
14131	11771	Ancient	-13.14	17.70	43.6	16.6	3.06	10.7
14140	11772	Ancient	-13.46	17.17	43.7	16.5	3.09	9.6
14145	11773	Ancient	-14.22	19.08	44.1	16.9	3.05	13.6
14146	11774	Ancient	-14.15	18.45	44.2	16.8	3.07	13.2
14147	11775	Ancient	-13.34	16.99	42.4	16.1	3.08	9.9
14149	11776	Ancient	-13.29	18.11	43.0	16.8	2.99	11.6
14151	11777	Ancient	-14.02	17.54	44.1	17.1	3.00	11.2
**LE/HCl (Collagen)**								
11500	11548	Modern	-22.52	5.56	43.1	16.0	3.13	13.5
11501	11549	Modern	-17.74	5.93	43.3	16.1	3.15	14.6
11502	11550	Modern	-19.07	11.15	43.0	16.1	3.12	12.6
11503	11551	Modern	-19.48	10.52	43.0	16.0	3.14	13.3
11504	11552	Modern	-21.75	12.06	42.7	15.7	3.16	12.7
11506	11554	Modern	-16.85	9.02	42.8	16.0	3.12	16.9
11507	11555	Modern	-17.19	11.72	42.6	15.8	3.15	15.0
11508	11556	Modern	-17.20	8.91	42.4	15.8	3.13	14.9
11509	11557	Modern	-17.71	11.11	42.3	15.8	3.13	15.2

11510	11558	Modern	-18.67	9.43	42.7	16.0	3.11	16.3
11511	11559	Modern	-17.28	11.71	42.6	15.9	3.12	13.0
11512	11560A	Modern	-16.11	11.74	42.7	15.9	3.14	14.9
	11560B	Modern	-16.33	11.77	43.2	16.2	3.11	17.3
	11560C	Modern	-16.13	11.82	43.2	16.1	3.13	15.0
	11560D	Modern	-15.99	11.49	43.4	16.9	3.00	16.1
	11560E	Modern	-15.88	11.46	43.1	16.9	2.98	15.6
11513	11561A	Modern	-18.51	10.56	45.0	17.0	3.09	14.9
	11561B	Modern	-18.60	10.60	45.5	17.0	3.13	16.2
	11561C	Modern	-18.50	10.52	45.3	17.0	3.10	14.3
	11561D	Modern	-18.76	10.53	46.1	17.2	3.12	14.5
	11561E	Modern	-18.49	10.58	44.6	16.8	3.11	14.9
11514	11562A	Modern	-19.84	4.65	44.7	16.1	3.23	8.0
	11562B	Modern	-20.07	4.33	44.0	15.9	3.23	9.4
	11562C	Modern	-19.83	4.72	43.7	15.6	3.26	9.0
	11562D	Modern	-19.75	4.79	43.9	15.7	3.26	9.6
	11562E	Modern	-19.69	4.59	44.3	15.8	3.28	9.2
11515	11563A	Modern	-22.16	5.11	44.9	16.8	3.13	13.7
	11563B	Modern	-21.57	5.25	45.3	16.9	3.13	14.5
	11563C	Modern	-21.99	5.12	44.7	16.61	3.14	13.6
	11563D	Modern	-21.98	5.14	45.6	16.9	3.15	13.8
	11563E	Modern	-21.81	5.14	45.6	17.0	3.14	13.3
11516	11564A	Modern	-22.24	5.78	44.5	16.6	3.13	12.9
	11564B	Modern	-22.07	5.74	44.9	16.7	3.15	13.1
	11564C	Modern	-22.01	5.68	45.7	16.9	3.16	12.6
	11564D	Modern	-22.2	5.66	45.0	16.7	3.15	12.2
	11564E	Modern	-21.78	5.81	44.9	16.6	3.15	13.1
11517	11565	Modern	-14.62	6.19	44.5	16.6	3.12	14.3
11518	11566	Modern	-17.76	6.41	46.5	17.3	3.13	17.8
10788	11567	Modern	-17.37	12.04	45.8	17.1	3.13	15.6
10787	11568	Modern	-17.75	9.94	44.8	16.4	3.19	14.2
10786	11569	Modern	-21.76	9.31	44.7	16.6	3.15	12.7
10785	11570	Modern	-20.83	7.40	44.2	16.4	3.15	12.4
10784	11571	Modern	-16.02	11.11	45.2	16.7	3.16	13.7
10263	11572	Modern	-12.93	7.01	44.5	16.5	3.14	12.1
10247	11573	Modern	-21.02	6.19	45.9	16.9	3.16	17.4
3619	11574	Modern	-15.18	6.06	45.9	17.1	3.12	17.5
1284	11575	Modern	-23.07	3.61	45.2	17.0	3.11	18.1
1260	11576	Modern	-22.98	3.30	45.3	16.9	3.12	18.5
12092	11577	Modern	-21.06	6.37	45.0	16.6	3.16	13.9
13003	11718	Ancient	-13.86	17.18	41.6	15.1	3.22	13.1
13005	11719	Ancient	-13.64	17.08	41.1	15.2	3.18	12.4
13008	11720	Ancient	-12.85	16.95	40.5	15.0	3.17	13.3
13009	11721	Ancient	-13.76	16.71	39.6	14.3	3.24	13.0
13010	11722	Ancient	-13.45	15.88	39.4	14.3	3.22	11.3
13012	11723	Ancient	-14.85	18.76	39.3	14.3	3.20	7.8
13015	11724	Ancient	-13.72	16.90	40.8	15.0	3.17	13.9
13019	11725	Ancient	-13.55	16.51	40.3	15.0	3.15	12.0
14080	11726	Ancient	-13.55	16.95	42.6	15.8	3.14	15.1
14082	11727	Ancient	-15.02	19.21	41.5	15.5	3.12	17.6
14087	11728	Ancient	-13.08	18.75	43.7	16.1	3.17	15.9
14089	11729	Ancient	-13.69	17.60	43.3	16.1	3.14	14.9
14091	11730	Ancient	-13.96	16.80	40.8	15.3	3.12	14.6
14097	11731	Ancient	-14.01	18.10	37.9	16.0	3.17	15.3
14105	11732	Ancient	-13.07	19.08	42.9	15.9	3.15	13.6
14106	11733	Ancient	-13.86	17.44	40.9	15.2	3.13	14.6

14108	11734	Ancient	-13.85	18.64	42.7	15.9	3.14	14.4
14112	11735	Ancient	-13.82	19.08	42.7	16.1	3.09	14.6
14113	11736	Ancient	-13.67	18.40	43.3	16.3	3.10	14.5
14120	11737	Ancient	-13.73	17.62	42.7	16.0	3.12	14.4
14121	11738	Ancient	-13.32	16.99	41.7	15.5	3.14	13.3
14125	11739	Ancient	-13.74	19.39	43.4	16.1	3.15	14.8
14127	11740	Ancient	-13.72	17.83	42.5	15.7	3.15	15.2
14131	11741	Ancient	-13.13	17.68	41.7	15.6	3.13	15.6
14140	11742	Ancient	-13.62	17.33	42.6	15.9	3.13	14.0
14145	11743	Ancient	-14.27	18.60	46.1	16.7	3.22	16.2
14146	11744	Ancient	-14.35	18.34	45.4	16.3	3.26	14.9
14147	11745	Ancient	-13.34	16.92	42.5	15.3	3.24	14.2
14149	11746	Ancient	-13.35	17.30	41.7	15.3	3.17	15.6
14151	11747	Ancient	-13.94	17.50	43.7	16.4	3.11	15.3
**LE/0 (Whole Bone)**								
11500	11608†	Modern	-22.26	5.35	14.0	9.2	1.77	N/A
11501	11610	Modern	-17.47	5.74	13.9	4.3	3.75	N/A
11502	11612	Modern	-18.54	10.82	12.6	3.8	3.88	N/A
11503	11614	Modern	-18.74	10.08	12.8	3.8	3.89	N/A
11504	11616	Modern	-21.65	11.54	12.0	3.5	4.02	N/A
11506	11620	Modern	-16.98	8.58	13.1	4.0	3.87	N/A
11507	11622	Modern	-17.10	11.28	12.3	3.6	3.94	N/A
11508	11624	Modern	-18.03	8.60	12.8	3.9	3.86	N/A
11509	11626	Modern	-17.27	10.26	12.1	3.6	3.92	N/A
11510	11628	Modern	-18.79	8.73	13.4	4.1	3.85	N/A
11511	11630	Modern	-16.93	10.84	11.9	3.6	3.91	N/A
11517	11637	Modern	-14.31	6.25	13.8	4.6	3.51	N/A
11518	11639	Modern	-16.94	6.16	14.7	4.8	3.59	N/A
10788	11641	Modern	-17.37	11.53	13.3	4.3	3.57	N/A
13003	11778	Ancient	-14.25	16.76	9.4	2.7	4.10	N/A
13005	11780	Ancient	-14.43	17.29	10.3	3.1	3.82	N/A
13008	11782	Ancient	-13.27	17.12	9.7	3.0	3.75	N/A
13009	11784	Ancient	-14.12	17.38	9.1	2.7	3.91	N/A
13010	11786	Ancient	-13.65	16.24	8.6	2.6	3.80	N/A
13012	11788	Ancient	-15.09	19.01	9.3	2.6	4.14	N/A
13015	11790	Ancient	-13.99	17.05	9.2	2.6	4.06	N/A
13019	11792	Ancient	-13.85	16.66	9.3	2.9	3.82	N/A
14080	11794	Ancient	-13.89	17.39	11.1	3.4	3.77	N/A
14082	11796	Ancient	-15.31	19.88	12.1	3.9	3.59	N/A
14087	11798	Ancient	-13.84	19.59	12.7	3.9	3.83	N/A
14089	11800	Ancient	-13.69	17.86	11.2	3.4	3.84	N/A
14091	11802	Ancient	-14.32	17.47	10.6	3.3	3.77	N/A
14097	11804	Ancient	-14.1	18.44	11.5	3.6	3.68	N/A
14105	11806	Ancient	-13.22	19.47	10.4	3.2	3.81	N/A
**0/0 (Whole Bone)**								
11500	11609	Modern	-23.33	5.28	15.9	4.2	4.48	N/A
11501	11611	Modern	-18.26	5.80	15.2	4.4	4.07	N/A
11502	11613	Modern	-20.3	10.84	14.6	3.8	4.45	N/A
11503	11615	Modern	-19.68	10.39	14.1	3.8	4.30	N/A
11504	11617	Modern	-22.43	11.64	13.5	3.5	4.54	N/A
11506	11621	Modern	-19.39	8.46	14.6	3.8	4.51	N/A
11507	11623	Modern	-18.35	11.14	13.7	3.7	4.35	N/A
11508	11625	Modern	-18.91	8.51	14.2	3.8	4.35	N/A
11509	11627	Modern	-18.23	10.25	13.4	3.6	4.28	N/A
11510	11629	Modern	-20.05	8.89	15.4	4.0	4.43	N/A
11511	11631	Modern	-18.03	10.97	13.0	3.4	4.45	N/A

11512	11632A	Modern	-17.29	11.19	14.7	3.9	4.39	N/A
	11632B	Modern	-17.04	11.44	14.5	3.9	4.38	N/A
	11632C	Modern	-17.11	11.36	14.7	3.9	4.44	N/A
	11632D	Modern	-17.08	11.26	14.6	3.9	4.37	N/A
	11632E	Modern	-17.21	11.25	14.7	3.9	4.38	N/A
11513	11633A	Modern	-19.66	10.37	14.8	3.8	4.54	N/A
	11633B	Modern	-19.69	10.25	14.7	3.8	4.53	N/A
	11633C	Modern	-19.82	10.35	14.8	3.8	4.59	N/A
	11633D	Modern	-19.49	10.34	14.6	3.8	4.51	N/A
	11633E	Modern	-19.77	10.43	15.0	3.9	4.52	N/A
11514	11634A	Modern	-20.73	4.86	18.0	3.9	5.44	N/A
	11634B	Modern	-20.70	4.83	18.1	3.9	5.37	N/A
	11634C	Modern	-20.63	4.76	17.8	3.9	5.37	N/A
	11634D	Modern	-20.61	4.92	17.9	3.9	5.40	N/A
	11634E	Modern	-20.69	4.82	17.9	3.9	5.42	N/A
11515	11635A	Modern	-21.99	5.06	14.9	4.1	4.22	N/A
	11635B	Modern	-21.96	4.92	14.9	4.1	4.20	N/A
	11635C†	Modern	-21.82	4.88	14.9	8.4	2.06	N/A
	11635D	Modern	-21.95	4.65	14.9	4.2	4.13	N/A
	11635E	Modern	-22.08	4.70	14.9	4.2	4.16	N/A
11516	11636A	Modern	-21.88	5.51	13.9	3.9	4.16	N/A
	11636B	Modern	-22.03	5.57	13.7	3.9	4.14	N/A
	11636C	Modern	-22.07	5.63	13.7	3.9	4.09	N/A
	11636D	Modern	-21.81	5.65	13.7	3.9	4.09	N/A
	11636E	Modern	-21.71	5.68	13.5	3.9	4.06	N/A
11517	11638	Modern	-14.78	5.62	15.7	4.6	4.00	N/A
11518	11640	Modern	-16.58	6.43	18.2	4.7	4.54	N/A
10788	11642	Modern	-17.30	11.77	13.6	4.3	3.68	N/A
10787	11643	Modern	-18.00	9.18	11.4	3.3	4.04	N/A
10786	11644	Modern	-22.32	8.80	13.0	3.6	4.18	N/A
10785	11645	Modern	-22.02	6.76	12.8	3.5	4.32	N/A
10784	11646	Modern	-17.74	10.64	13.2	3.6	4.26	N/A
10263	11647	Modern	-13.24	6.99	15.6	4.6	3.93	N/A
10247	11648	Modern	-20.64	6.14	15.7	4.4	4.17	N/A
3619	11649	Modern	-16.41	5.54	13.7	4.4	3.66	N/A
1284	11650	Modern	-23.89	3.69	17.1	4.5	4.57	N/A
1260	11651	Modern	-24.61	3.49	20.0	4.8	4.91	N/A
12092	11652	Modern	-20.76	6.24	14.4	4.3	3.95	N/A
13003	11779	Ancient	-14.21	15.94	8.9	2.7	3.83	N/A
13005	11781	Ancient	-14.28	16.21	10.0	3.1	3.72	N/A
13008	11783	Ancient	-13.34	16.26	9.5	3.0	3.71	N/A
13009	11785	Ancient	-14.25	16.56	8.9	2.8	3.73	N/A
13010	11787	Ancient	-13.98	15.06	8.3	2.6	3.71	N/A
13012	11789	Ancient	-15.11	18.43	9.0	2.7	3.86	N/A
13015	11791	Ancient	-13.96	16.58	9.1	2.8	3.85	N/A
13019	11793	Ancient	-13.92	15.94	9.0	2.8	3.74	N/A
14080	11795	Ancient	-13.94	17.03	10.8	3.4	3.74	N/A
14082	11797	Ancient	-15.39	19.42	12.1	3.9	3.58	N/A
14087	11799	Ancient	-13.64	19.23	12.3	3.9	3.72	N/A
14089	11801	Ancient	-13.93	17.73	11.1	3.5	3.74	N/A
14091	11803	Ancient	-14.36	17.05	10.4	3.3	3.68	N/A
14097	11805	Ancient	-14.13	17.98	11.4	3.7	3.64	N/A
14105	11807	Ancient	-13.39	18.93	10.2	3.2	3.69	N/A
14106	11808	Ancient	-14.22	17.22	10.8	3.5	3.64	N/A
14108	11809	Ancient	-14.34	18.42	11.0	3.6	3.58	N/A
14112	11810	Ancient	-14.25	18.51	11.6	3.5	3.89	N/A

14113	11811	Ancient	-14.21	18.49	11.6	3.4	3.97	N/A
14120	11812	Ancient	-13.88	18.11	11.0	3.4	3.75	N/A
14121	11813	Ancient	-13.68	17.59	10.7	3.4	3.67	N/A
14125	11814	Ancient	-14.29	19.66	11.7	3.6	3.79	N/A
14127	11815	Ancient	-14.28	18.12	11.6	3.5	3.88	N/A
14131	11816	Ancient	-13.47	17.72	11.2	3.4	3.82	N/A
14140	11817	Ancient	-13.62	17.30	10.7	3.2	3.90	N/A
14145	11818	Ancient	-14.38	18.64	12.4	3.7	3.87	N/A
14146	11819	Ancient	-14.48	19.43	12.0	3.8	3.66	N/A
14147	11820	Ancient	-13.71	17.84	10.7	3.3	3.82	N/A
14149	11821	Ancient	-13.37	18.22	11.4	3.8	3.55	N/A
14151	11822	Ancient	-14.06	17.56	12.1	3.8	3.72	N/A

†denotes a sample that had extremely unusual elemental composition and was therefore excluded from the statistical tests given the likelihood of error during the elemental and isotopic measurements.

Statistical Results for Elemental Data

The following table contains the average elemental data for different species examined

Table S8. Average elemental compositions of different taxa across different treatments

Species	n		Expected†	LE/HCl	LE/EDTA	0/0	LE/0
Kangaroo (modern)	16	wt% C	42.2±0.3	43.6±1.2	43.3±0.6	13.7±1.0	12.6±0.5
		wt% N	15.3±0.1	16.2±0.4	17.0±0.2	3.7±0.3	3.8±0.3
		Atomic C:N	3.22±0.05	3.10±0.03	2.97±0.01	4.31±0.21	3.87±0.12
Cow (modern)	11	wt% C	42.2±0.3	45.1± 0.7	44.0±0.6	15.9±1.9	14.2±0.5
		wt% N	15.3±0.1	16.8±0.3	17.1±0.3	4.4±0.3	4.6±0.2
		Atomic C:N	3.22±0.05	3.14±0.01	2.99±0.01	4.19±0.35	3.62± 0.12
Ringed Seal (ancient)	30	wt% C	42.2±0.3	41.5±1.7	42.4± 1.4	10.8±1.2	10.3±1.2
		wt% N	15.3±0.1	15.4±0.6	15.9±0.5	3.3±0.4	3.1±0.5
		Atomic C:N	3.22±0.05	3.13±0.04	3.12 ±0.04	3.81±0.10	3.85±0.15

†Based on amino acid sequence data from modern mammalian collagen (Guiry & Szpak 2020)

The following tables contain data from the paired t-tests comparing wt% C and wt% N of the various treatments performed.

Table S9. *p* value results for Mann-Whitney U tests comparing wt% N between treatments. Bolded values represent comparisons yielding *p* values below the alpha threshold (0.05)

Samples	*df*	Treatment	LE/HCl	LE/EDTA	LE/0
Modern	48	LE/EDTA	**<0.001**	—	—
		LE/0	**<0.001**	**<0.001**	—
		0/0	**<0.001**	**<0.001**	0.93

Ancient	29	LE/EDTA<br>LE/0<br>0/0	**0.02**<br>**<0.001**<br>**<0.001**	—<br>**<0.001**<br>**<0.001**	—<br>—<br>0.85

Table S10. *p* value results for Mann-Whitney U tests comparing wt% C between treatments. Bolded values represent comparisons yielding *p* values below the alpha threshold (0.05)

Samples	*df*	Treatment	LE/HCl	LE/EDTA	LE/0
Modern	48	LE/EDTA<br>LE/0<br>0/0	**<0.001**<br>**<0.001**<br>**<0.001**	—<br>**<0.001**<br>**<0.001**	—<br>—<br>**0.001**
Ancient	29	LE/EDTA<br>LE/0<br>0/0	0.07<br>**<0.001**<br>**<0.001**	—<br>**<0.001**<br>**<0.001**	—<br>—<br>0.66

Collagen Yield Results

For the modern samples, there were no significant differences in the collagen yield (wt% collagen relative to starting bone mass) for EDTA-treated and HCl-treated samples ($p = 0.10$). The average yield for modern HCl-treated samples was 14.1%, while the yield for modern EDTA-treated samples was 14.5% (Table S11). The ancient samples did, however, display a significant difference in collagen yield between EDTA and HCl-treated samples ($p < 0.001$). On average, the yields for EDTA-treated samples (11.0%) were lower than the yields for HCl-treated samples (14.2%) (Table S11).

The following table contains the average yield of collagen prepared using two different demineralization treatments:

Table S11. Average collagen yields for modern and ancient samples by treatment

Sample Context	Treatment	Average Sample Yield	*p* value for Mann- Whitney U comparison
Modern (*n=49*)	LE/HCl	14.1%	0.26
	LE/EDTA	14.5%	
Ancient (*n=29*)	LE/HCl	14.2%	**<0.001**
	LE/EDTA	11.0%	

Amino Acid Composition Data

The following tables contain amino acid composition data from a subset of the modern and ancient samples used for isotopic and elemental analysis. The analyses were performed in Toronto, Ontario, Canada at the SPARC BioCenter at the Hospital for Sick Children. The number of amino acid residues per 1000 residues was calculated using the molar mass of each amino acid and the number of grams of that particular amino acid per 100g of the total protein.

Table S12. Comparison of the amino acid composition of a subset of modern samples treated with HCl and EDTA

**HCl/0**				**EDTA/0**			
Sample Number	Amino Acid	g/100g	Approximate # aa/1000aa	Sample Number	Amino Acid	g/100g	Approximate # aa/1000aa

11556	Asparagine/Aspartic Acid	4.306	51	11586	Asparagine/Aspartic Acid	4.383	50
	Glutamine/Glutamic Acid	7.633	82		Glutamine/Glutamic Acid	7.930	81
	Hydroxyproline	7.054	85		Hydroxyproline	7.516	86
	Serine	2.502	38		Serine	2.606	37
	Glycine	14.585	308		Glycine	15.506	312
	Histidine	0.824	8		Histidine	0.867	8
	Arginine	6.179	56		Arginine	6.516	56
	Threonine	1.447	19		Threonine	1.472	19
	Alanine	6.079	108		Alanine	6.391	108
	Proline	8.739	120		Proline	9.260	121
	Tyrosine	0.669	6		Tyrosine	0.683	6
	Valine	1.934	26		Valine	1.982	26
	Methionine	0.567	6		Methionine	0.693	7
	Isoleucine	0.743	9		Isoleucine	0.759	9
	Leucine	2.280	28		Leucine	2.337	27
	Phenylalanine	1.801	17		Phenylalanine	1.828	17
	Lysine	2.844	31		Lysine	2.892	30
11555	Asparagine/Aspartic Acid	4.362	52	11585	Asparagine/Aspartic Acid	4.453	50
	Glutamine/Glutamic Acid	7.734	83		Glutamine/Glutamic Acid	7.995	82
	Hydroxyproline	7.274	88		Hydroxyproline	7.666	88
	Serine	2.544	38		Serine	2.603	37
	Glycine	14.762	312		Glycine	15.466	311
	Histidine	0.876	9		Histidine	0.880	9
	Arginine	6.263	57		Arginine	6.530	57
	Threonine	1.474	20		Threonine	1.476	19
	Alanine	6.167	110		Alanine	6.439	109
	Proline	8.706	120		Proline	9.023	118
	Tyrosine	0.709	6		Tyrosine	0.684	6
	Valine	1.968	27		Valine	1.972	25
	Methionine	0.667	7		Methionine	0.685	7
	Isoleucine	0.766	9		Isoleucine	0.762	9
	Leucine	2.346	28		Leucine	2.350	27
	Phenylalanine	1.826	18		Phenylalanine	1.914	17
	Lysine	2.758	30		Lysine	2.812	29
11552	Asparagine/Aspartic Acid	4.389	52	11582	Asparagine/Aspartic Acid	4.168	47
	Glutamine/Glutamic Acid	7.785	84		Glutamine/Glutamic Acid	7.497	77
	Hydroxyproline	7.270	88		Hydroxyproline	7.053	81
	Serine	2.582	39		Serine	2.440	35
	Glycine	14.911	315		Glycine	14.457	291
	Histidine	0.917	9		Histidine	0.823	8
	Arginine	6.314	57		Arginine	6.096	53

	Threonine	1.485	20		Threonine	1.405	18
	Alanine	6.176	110		Alanine	6.018	102
	Proline	8.920	123		Proline	8.540	112
	Tyrosine	0.739	6		Tyrosine	0.636	5
	Valine	1.989	27		Valine	1.861	24
	Methionine	0.656	7		Methionine	0.653	7
	Isoleucine	0.789	10		Isoleucine	0.726	8
	Leucine	2.384	29		Leucine	2.215	25
	Phenylalanine	1.919	18		Phenylalanine	1.687	15
	Lysine	2.759	30		Lysine	2.604	27

Table S13. Comparison of the amino acid composition of a subset of ancient samples treated with HCl and EDTA

HCl/0				EDTA/0			
Sample Number	Amino Acid	g/100g	Approximate # aa/1000aa	Sample Number	Amino Acid	g/100g	Approximate # aa/1000aa
11723	Asparagine/Aspartic Acid	3.623	50	11753	Asparagine/Aspartic Acid	3.409	45
	Glutamine/Glutamic Acid	6.662	83		Glutamine/Glutamic Acid	6.652	80
	Hydroxyproline	6.280	88		Hydroxyproline	6.896	93
	Serine	2.387	42		Serine	2.42	41
	Glycine	12.571	307		Glycine	13.66	320
	Histidine	0.846	10		Histidine	0.771	9
	Arginine	5.335	56		Arginine	5.576	56
	Threonine	1.424	22		Threonine	1.364	20
	Alanine	4.765	98		Alanine	5.073	100
	Proline	7.521	120		Proline	8.017	122
	Tyrosine	0.623	6		Tyrosine	0.442	4
	Valine	1.856	29		Valine	1.746	26
	Methionine	0.596	7		Methionine	0.614	7
	Isoleucine	0.713	10		Isoleucine	0.672	9
	Leucine	2.061	29		Leucine	1.922	26
	Phenylalanine	1.485	16		Phenylalanine	1.523	16
	Lysine	2.154	27		Lysine	2.137	26
11722	Asparagine/Aspartic Acid	3.645	50	11752	Asparagine/Aspartic Acid	3.228	43
	Glutamine/Glutamic Acid	6.547	82		Glutamine/Glutamic Acid	6.259	75
	Hydroxyproline	6.400	89		Hydroxyproline	6.484	87
	Serine	2.375	41		Serine	2.272	38
	Glycine	12.700	310		Glycine	12.764	299
	Histidine	0.761	9		Histidine	0.681	8
	Arginine	5.340	56		Arginine	5.233	53
	Threonine	1.402	22		Threonine	1.284	19

	Alanine	4.751	98		Alanine	4.732	93
	Proline	7.569	121		Proline	7.442	114
	Tyrosine	0.615	6		Tyrosine	0.421	4
	Valine	1.822	29		Valine	1.628	24
	Methionine	0.548	7		Methionine	0.54	6
	Isoleucine	0.714	10		Isoleucine	0.652	9
	Leucine	2.002	28		Leucine	1.791	24
	Phenylalanine	1.510	17		Phenylalanine	1.324	14
	Lysine	2.089	26		Lysine	2.005	24
11721	Asparagine/Aspartic Acid	3.679	51	11751	Asparagine/Aspartic Acid	3.402	45
	Glutamine/Glutamic Acid	6.670	83		Glutamine/Glutamic Acid	6.662	80
	Hydroxyproline	6.546	92		Hydroxyproline	6.902	93
	Serine	2.418	42		Serine	2.432	41
	Glycine	13.052	319		Glycine	13.755	322
	Histidine	0.842	10		Histidine	0.790	9
	Arginine	5.444	57		Arginine	5.593	56
	Threonine	1.415	22		Threonine	1.371	20
	Alanine	4.897	101		Alanine	5.089	100
	Proline	7.823	125		Proline	8.089	124
	Tyrosine	0.558	6		Tyrosine	0.437	4
	Valine	1.811	28		Valine	1.740	26
	Methionine	0.561	7		Methionine	0.572	7
	Isoleucine	0.735	10		Isoleucine	0.712	10
	Leucine	2.004	28		Leucine	1.910	26
	Phenylalanine	1.605	18		Phenylalanine	1.454	15
	Lysine	2.133	27		Lysine	2.112	25

Table S14. Comparison of the atomic C:N ratio calculated by EA-IRMS and UPLC of paired samples treated with HCl and EDTA. Rows shaded in light blue correspond to modern samples, while unshaded rows correspond to ancient samples.

**HCl/0**			**EDTA/0**		
Sample Number	Atomic C:N Ratio by EA-IRMS	Atomic C:N Ratio by Amino Acid Composition †	Sample Number	Atomic C:N Ratio by EA-IRMS	Atomic C:N Ratio by Amino Acid Composition †
11556	3.13	2.92	11586	2.96	2.91
11555	3.15	2.92	11585	2.97	2.91
11552	3.16	2.93	11582	2.97	2.91
11723	3.18	2.93	11753	3.09	2.92
11722	3.14	2.93	11752	3.09	2.91
11721	3.15	2.93	11751	3.10	2.91

† Atomic C:N ratio by amino acid composition is calculated under the assumption that no deamidation is occurring to asparagine or glutamine

Practical Considerations when Choosing a Demineralization Agent

Beyond the potential effects that demineralization agents have on the stable isotope and elemental composition of bone collagen, there are additional considerations that should be taken into account when assessing the efficacy of different demineralization methods. Firstly, HCl solution is less costly to prepare than EDTA, and demineralization using HCl proceeds much more quickly than equivalent samples treated with EDTA (Collins & Galley 1998). Additionally, preparing an EDTA solution is more complex and time consuming than preparing an equivalent HCl solution. Due to these differences in cost and solution preparation, most laboratory groups opt for the HCl demineralization. Regardless of the potential drawbacks of using EDTA as a demineralization agent, it may be very useful in cases where ancient DNA analysis will be performed, given that this agent does not impact DNA integrity (Hagelberg & Clegg 1991).

References

Ambrose SH. 1990. Preparation and characterization of bone and tooth collagen for isotopic analysis. *Journal of Archaeological Science* 17:431-451. 10.1016/0305-4403(90)90007-R

Collins M, and Galley P. 1998. Towards an optimal method of archaeological collagen extraction: the influence of pH and grinding. *Ancient Biomolecules* 2:209-223.

DeNiro MJ. 1985. Postmortem preservation and alteration of *in vivo* bone collagen isotope ratios in relation to palaeodietary reconstruction. *Nature* 317:806-809. 10.1038/317806a0

Guiry EJ, and Szpak P. 2020. Quality control for modern bone collagen stable carbon and nitrogen isotope measurements. *Methods in Ecology and Evolution* 11:1049-1060.

Hagelberg E, and Clegg JB. 1991. Isolation and characterization of DNA from archaeological bone. *Proceedings of the Royal Society of London Series B: Biological Sciences* 244:45-50.

Schimmelmann A, Qi H, Coplen TB, Brand WA, Fong J, Meier-Augenstein W, Kemp HF, Toman B, Ackermann A, and Assonov S. 2016. Organic reference materials for hydrogen, carbon, and nitrogen stable isotope-ratio measurements: caffeines, n-alkanes, fatty acid methyl esters, glycines, L-valines, polyethylenes, and oils. *Analytical chemistry* 88:4294-4302.

Szpak P, Metcalfe JZ, and Macdonald RA. 2017. Best Practices for Calibrating and Reporting Stable Isotope Measurements in Archaeology. *Journal of Archaeological Science: Reports* 13:609-616. 10.1016/j.jasrep.2017.05.007
