# Supplementary figures and images for: Acidification does not alter the stable isotope composition of bone collagen
Source: PeerJ. 2022 Jun 14;10:e13593. doi: 10.7717/peerj.13593 (PMC9205305; doi:10.7717/peerj.13593)

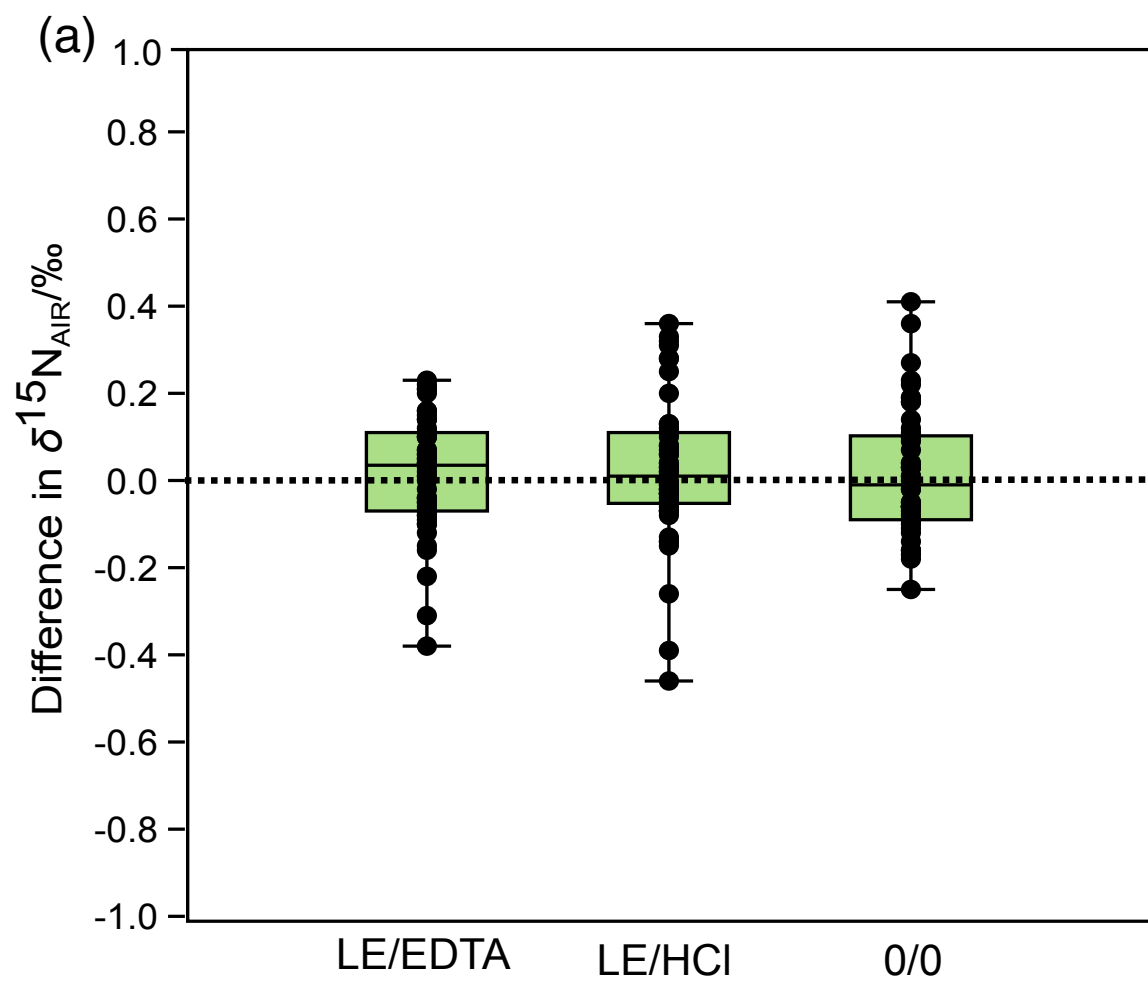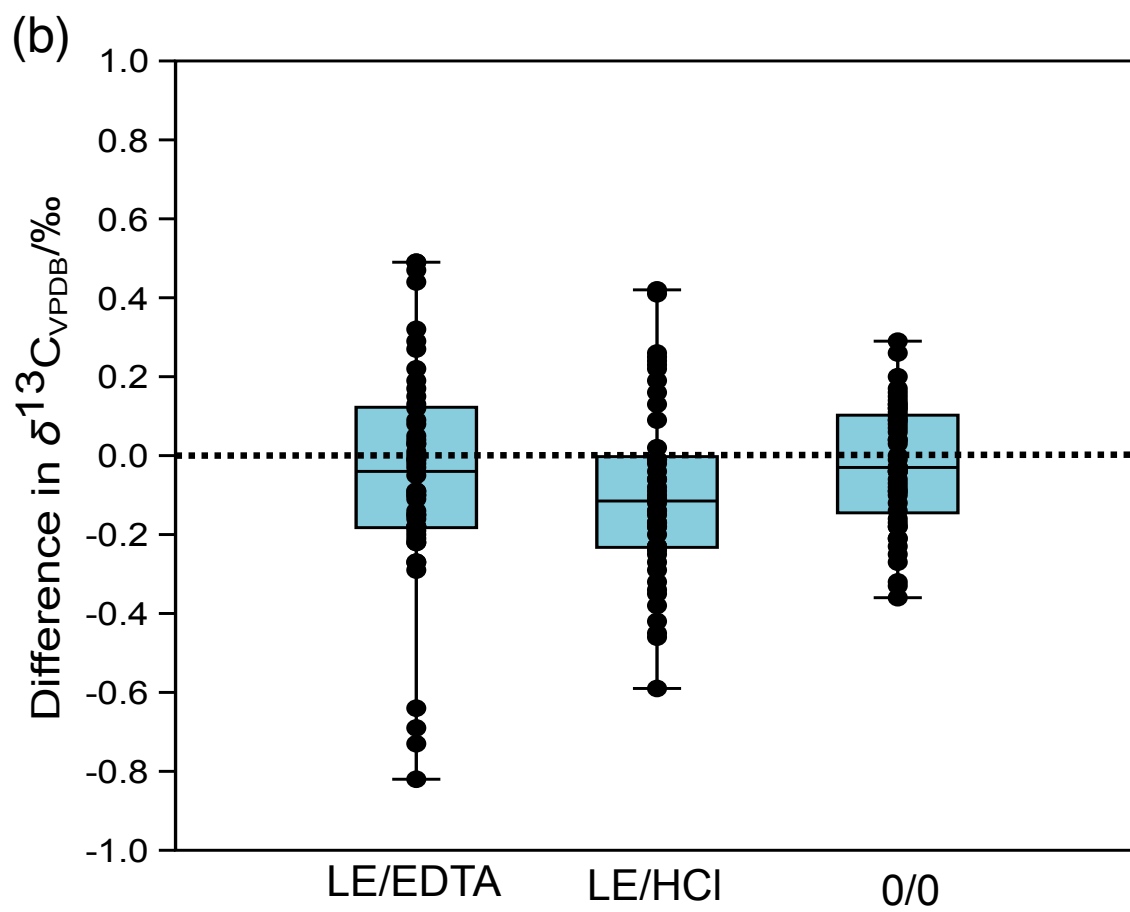

Supplement: Supplemental Information 2 — Boxplots depicting the difference in (A) δ15N, (B) δ13C values among replicates of the same samples. Differences between each of the replicates of the same samples were then pooled with all five samples from the same treatment. The dotted line represents a difference of zero. [file peerj-10-13593-s002.pdf]
